# Genome-wide identification and expression analysis of *bZIP* gene family in *Carthamus tinctorius* L.

**DOI:** 10.1038/s41598-020-72390-z

**Published:** 2020-09-23

**Authors:** Haoyang Li, Lixia Li, Guodong ShangGuan, Chang Jia, Sinan Deng, Muhammad Noman, Yilin Liu, Yongxin Guo, Long Han, Xiaomei Zhang, Yuanyuan Dong, Naveed Ahmad, Linna Du, Haiyan Li, Jing Yang

**Affiliations:** College of Life Science, Engineering Research Center of the Chinese Ministry of Education for Bioreactor and Pharmaceutical Development, Jilin Agricultural University, Changchun, 130118 China

**Keywords:** Nanobiotechnology, Plant biotechnology

## Abstract

The basic leucine zipper (bZIP) is a widely known transcription factors family in eukaryotes. In plants, the role of bZIP proteins are crucial in various biological functions such as plant growth and development, seed maturation, response to light signal and environmental stress. To date, bZIP protein family has been comprehensively identified in *Arabidopsis*, castor, rice, ramie, soybean and other plant species, however, the complete genome-wide investigation of *Carthamus tinctorius*-bZIP family still remains unexplained. Here, we identified 52 putative *bZIP* genes from *Carthamus tinctorius* using a draft genome assembly and further analyzed their evolutionary classification, physicochemical properties, Conserved domain analysis, functional differentiation and the investigation of expression level in different tissues. Based on the common bZIP domain, CtbZIP family were clustered into 12 subfamilies renamed as (A–J, S, X), of which the X is a unique subfamily to *Carthamus tinctorius*. A total of 20 conserved protein motifs were found in CtbZIP proteins. The expression profiling of *CtbZIP* genes deciphered their tissue-specific pattern. Furthermore, the changes in CtbZIP transcript abundance suggested that their transcription regulation could be highly influenced by light intensity and hormones. Collectively, this study highlights all functional and regulatory elements of bZIP transcription factors family in *Carthamus tinctorius* which may serve as potential candidates for functional characterization in future.

## Introduction

Transcription factors (TFs) are regulated through sequence-specific DNA-binding proteins which interact with relevant cis-acting elements^1^. The interaction alters transcription activity and stimulates or suppresses gene expression^2^. As switch of gene expression, TFs play important regulatory roles in almost all processes of plant life^3^. Functional characterization of TFs is in-depth analyzed through biological processes and transcriptional regulatory networks. Thus, TFs are significant components of abiotic stress signaling pathways. Among them, the basic leucine zipper (bZIP) also accounts for a large and diverse TF family. All bZIP TFs consist of two structural components: a basic region (N-x7-R/K-x9) for sequence-specific DNA binding, and a leucine zipper. In Arabidopsis, 78 *bZIP* genes have been reported^4^, whereas 89 in *Oryza sativa*^5^, 55 in *Vitis vinifera*^6^, 125 in *Zea mays*^7^, 247 in *Brassica napus L.*^8^, 92 in *Sorghum vulgare*^9^ and 131 in *Glycine max*^10^. In general, it is known that the putative *bZIP* genes have been classified as tens of groups on the basis of sequence similarities of their basic regions and conserved motifs. *AtbZIP* gene family was classified into 13 subfamilies in *Arabidopsis thaliana*^4^ while the predicted bZIP proteins of *Oryza sativa* into 11 groups based on DNA-binding specificity and amino acid sequence^11^. Although the number of groups of the *bZIP* family in *Oryza sativa* (11 groups) and *Arabidopsis thaliana* (13 groups) is different, however affiliation’s relationship is similar. The interspecies clustering also indicates that homologous bZIP from both species fell into common group^3^.

In plants, bZIP TFs regulate many transcriptional response pathways in multiple biological processes. They regulate the development of tissues and organs, including seed maturation and germination, embryogenesis^12^, blooming^13^ and photomorphogenesis^14^. In addition, bZIP TFs are involved in responses to abiotic and biotic stresses such as extreme temperatures, water deficit, high osmolarity and salinity and defence from pathogens^15,16^. AtbZIP TFs as TGA2, TGA5 and TGA6 regulate salicylic acid-dependent mechanisms and activate jasmonic acid and ethylene-dependent pathway in abiotic stress, while ABF3 and ABF4, play essential roles in ABA stress responses^17^. HY5 and HYH, the main regulators of photomorphogenesis, mediate the light response in *Arabidopsis thaliana*^18^. Similarly, OsBZ8 is induced by ABA and mediates salt resistance^19^, while LIP19 functions as a molecular switch for low-temperature signal transduction in rice^20^.

*Carthamus tinctorius* L. is an annual Asteraceae plant and an economic crop grown for edible oil extracted from its seed. It is suitable to grow in environments with insufficient moisture^21^ thus can be grown on marginal agricultural lands that are suitable for few other crops^20,21^. It is a minor oilseed crop in terms of production among unexploited crops, which not only has high ornamental value, but also has other important practical value. Safflower seeds are rich in oil and contain high levels of unsaturated fatty acids, vitamin E and oryzanol. The application of safflower seed oil is very extensive in fuel industry, cardiovascular health care and production of pharmaceuticals as a plant factory. Therefore, genetic improvement of safflower is necessary to increase its acceptability and utility as an important oilseed crop, but due to its limited gene information resource, this demand still persists. To accelerate the improvement of safflower, its genome was sequenced recently. Development of safflower gene resources has the potential to speed up the process of molecular breeding, and can be used to investigate patterns of genome evolution.

Complete genomic information of safflower has not yet been uncovered fully, therefore, demands the analysis of molecular mechanisms, network regulation and functional diversity. A draft genome assembly of safflower was produced covering 866 million bp after sequencing a single, short insert library to ~ 21 × depth^22^. The full-length transcriptome sequencing of safflower generated 10.43 GB clean data from which 38,302 redundant sequences were captured^23^. We accomplished the de novo transcriptome assembly of safflower from which we identified putative oleosin genes^24^ by Solexa-based deep sequencing and investigated some genes related to the biosynthesis of safflower yellow^25^. In addition, we have also sequenced the genome of safflower (Accession: PRJNA399628 ID: 399628). In this study, we screened fifty-two *bZIP* family genes from safflower genome database, named *CtbZIP1-52*. We not only analyzed the structural characteristics of CtbZIP family genes, identified CtbZIP motifs and constructed a phylogenetic tree, but also speculated their network regulating relationship and functional diversification among the members of CtbZIP family. The expression patterns of 52 CtbZIP genes in various tissues and different developmental stages were predicted by RPKM values, and the accuracy of expression profiles was verified by RT-qRCR. This study provides a comprehensive genome-wide investigation and expression analysis of CtbZIP family of safflower which would be important for functional characterization of CtbZIP TFs involved in biological processes and transcriptional regulatory networks, and then lay a foundation for molecular breeding of safflower in the future.

## Results

### Genome-wide identification of CtbZIP TFs

Through in silico analyses (detailed in methods), a total of 52 members in safflower *bZIP* gene family were identified. Based on the splicing results of genomic sequence, we sequentially sorted 52 CtbZIP proteins according to unigene number from small to large provisionality and named them CtbZIP1-52 (Table 1). Their name, ID, ORF (open reading frame) length and polypeptide length as well as conserved domain position are mentioned in Table 1 while molecular weight, PI (Isoelectric Point) and Grand Average of Hydropathicity (GRAVY) are given in supplementary file S1, Table S1. The predicted molecular weights lie between 11.98 kDa to 86.19 kDa while PI ranges from 4.86 to 9.78. *CtbZIP38* gene has the shortest conserved domain with 21 amino acids, whereas *CtbZIP10* possesses the longest domain (74 amino acids). All negative GRAVY values indicate their hydrophilic nature.

**Table 1:** List of the identified safflower bZIP TFs and their attributes.

S. no	Name	ID in genomic data	Length of ORF	Number of aa	Conserved domain position (aa)
1	CtbZIP1	ccg000574	2052	683	197–258
2	CtbZIP2	ccg001746	795	264	186–229
3	CtbZIP3	ccg001897	981	326	238–284
4	CtbZIP4	ccg001910	1,113	370	240–277
5	CtbZIP5	ccg002216	1,050	349	107–168
6	CtbZIP6	ccg002309	516	171	25–82
7	CtbZIP7	ccg002770	852	283	236–278
8	CtbZIP8	ccg003603	1,101	366	289–338
9	CtbZIP9	ccg005204	594	197	71–130
10	CtbZIP10	ccg005666	945	314	178–224
11	CtbZIP11	ccg007675	1,044	347	150–223
12	CtbZIP12	ccg010618	810	269	90–148
13	CtbZIP13	ccg011035	891	296	138–188
14	CtbZIP14	ccg012241	1,710	569	525–550
15	CtbZIP15	ccg012409	1,302	433	354–405
16	CtbZIP16	ccg012979	1,152	383	295–357
17	CtbZIP17	ccg013975	1,218	405	41–92
18	CtbZIP18	ccg014366	1,119	372	243–281
19	CtbZIP19	ccg014560	567	188	75–133
20	CtbZIP20	ccg014897	339	112	57–127
21	CtbZIP21	ccg015074	429	142	39–88
22	CtbZIP22	ccg015568	318	105	34–85
23	CtbZIP23	ccg016238	426	141	56–96
24	CtbZIP24	ccg016704	1,422	473	257–309
25	CtbZIP25	ccg016850	471	156	44–95
26	CtbZIP26	ccg016950	477	158	48–108
27	CtbZIP27	ccg016979	1,167	388	252–314
28	CtbZIP28	ccg017531	780	259	165–222
29	CtbZIP29	ccg017533	750	249	165–209
30	CtbZIP30	ccg017757	465	154	33–77
31	CtbZIP31	ccg017772	417	138	27–85
32	CtbZIP32	ccg018261	1,245	414	309–371
33	CtbZIP33	ccg018785	1593	530	401–459
34	CtbZIP34	ccg018897	1509	502	365–409
35	CtbZIP35	ccg019734	1,041	346	56–95
36	CtbZIP36	ccg020071	444	147	24–63
37	CtbZIP37	ccg021349	519	172	55–108
38	CtbZIP38	ccg022221	639	212	171–191
39	CtbZIP39	ccg022423	459	152	65–116
40	CtbZIP40	ccg022869	1,560	519	215–256
41	CtbZIP41	ccg023166	1,257	418	216–268
42	CtbZIP42	ccg023405	969	322	241–280
43	CtbZIP43	ccg025170	1,005	334	201–245
44	CtbZIP44	ccg025235	1755	584	432–490
45	CtbZIP45	ccg027083	1,197	398	92–125
46	CtbZIP46	ccg028775	627	208	93–130
47	CtbZIP47	ccg029577	390	129	34–93
48	CtbZIP48	ccg030605	876	291	240–283
49	CtbZIP49	ccg031009	594	197	84–134
50	CtbZIP50	ccg031237	960	319	149–205
51	CtbZIP51	ccg031238	780	259	88–145
52	CtbZIP52	ccg031837	636	211	84–138

*ORF* open reading frame, *aa* amino acids.

### Classification of the CtbZIP proteins based on phylogram

**Figure 1:**
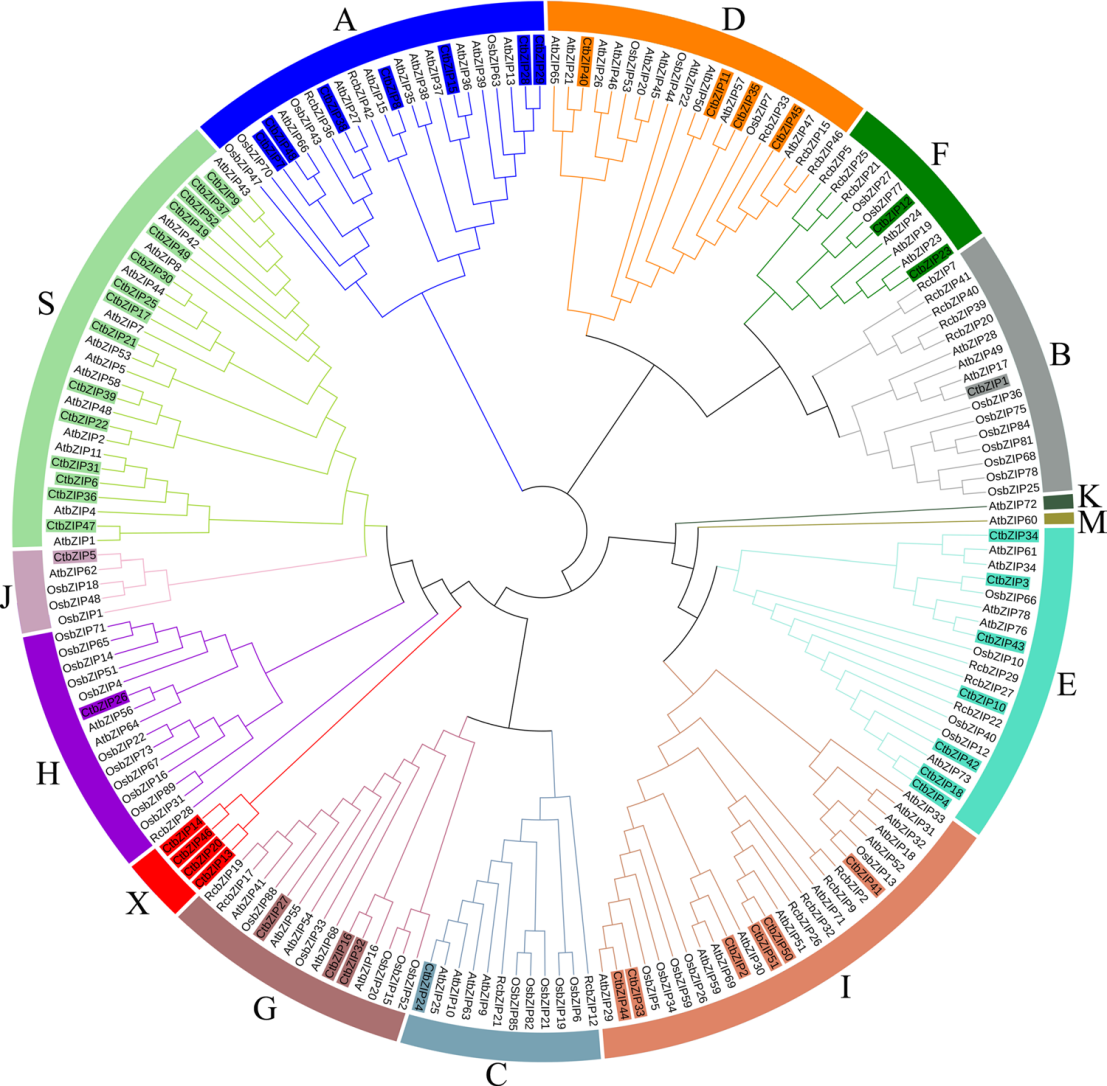
Cladogram of the CtbZIP TFs of Safflower, *Arabidopsis thaliana, Oryza sativa* and *Ricinus communis*. All bZIP TFs clustered into 12 distinct clades, marked by curves with different colors. Subfamily S has got the highest number (15) while J, H, C and B got the lowest (1) each, whereas 4 CtbZIPs exclusively clustered together in subfamily X.

We constructed a phylogenetic tree to elucidate the evolutionary relationship among bZIP TFs of *Carthamus tinctorius* L., *Arabidopsis thaliana*, *Oryza sativa* and *Ricinus communis* (Fig. 1). *Arabidopsis thaliana* bZIP family has been classified into 13 subfamilies^4^. The bZIP TFs of most of plant species are classified according to the subfamilies of *Arabidopsis*. For example, the bZIP proteins of *Oryza sativa* were divided into 10 subfamilies^5^, *Ricinus communis* into 9^26^ and *Camellia sinensis* into 11^27^. We divided the 52 CtbZIP TFs into 12 subfamilies (CtbZIP-A, CtbZIP-B, CtbZIP-C, CtbZIP-D, CtbZIP-E, CtbZIP-F, CtbZIP-G, CtbZIP-H, CtbZIP-I, CtbZIP-J, CtbZIP-S and CtbZIP-X) on the basis of the classification of *Arabidopsis*. However, CtbZIP13, CtbZIP14, CtbZIP20 and CtbZIP46 could not be aggregated into any subfamily thus were grouped together into a separate branch named as subfamily X. None of CtbZIP proteins clustered into subfamily K and M indicating loss of these proteins throughout safflower evolution. A separate phylogenetic reconstruction elucidating the evolutionary relationship of Arabidopsis and safflower bZIP proteins is given in figure S1.

### Motif analysis of the CtbZIP proteins

**Figure 2:**
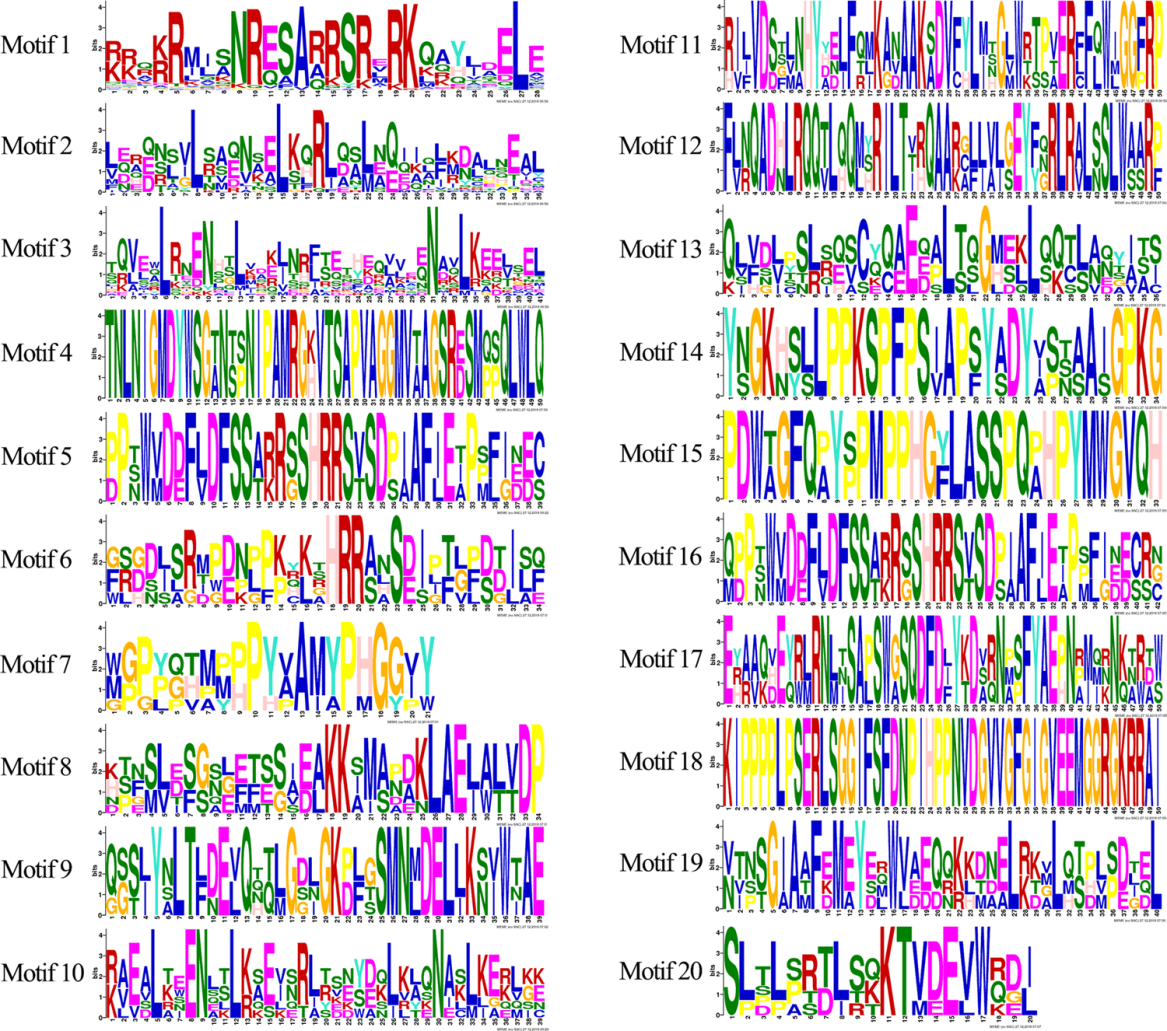
Sequence logos of CtbZIPs conserved motifs. From the multiply aligned protein sequences of CtbZIP, 20 conserved motifs were determined. Among them, Motif1 and Motif2 were common in all orthologs of the phylogenetic tree.

**Figure 3:**
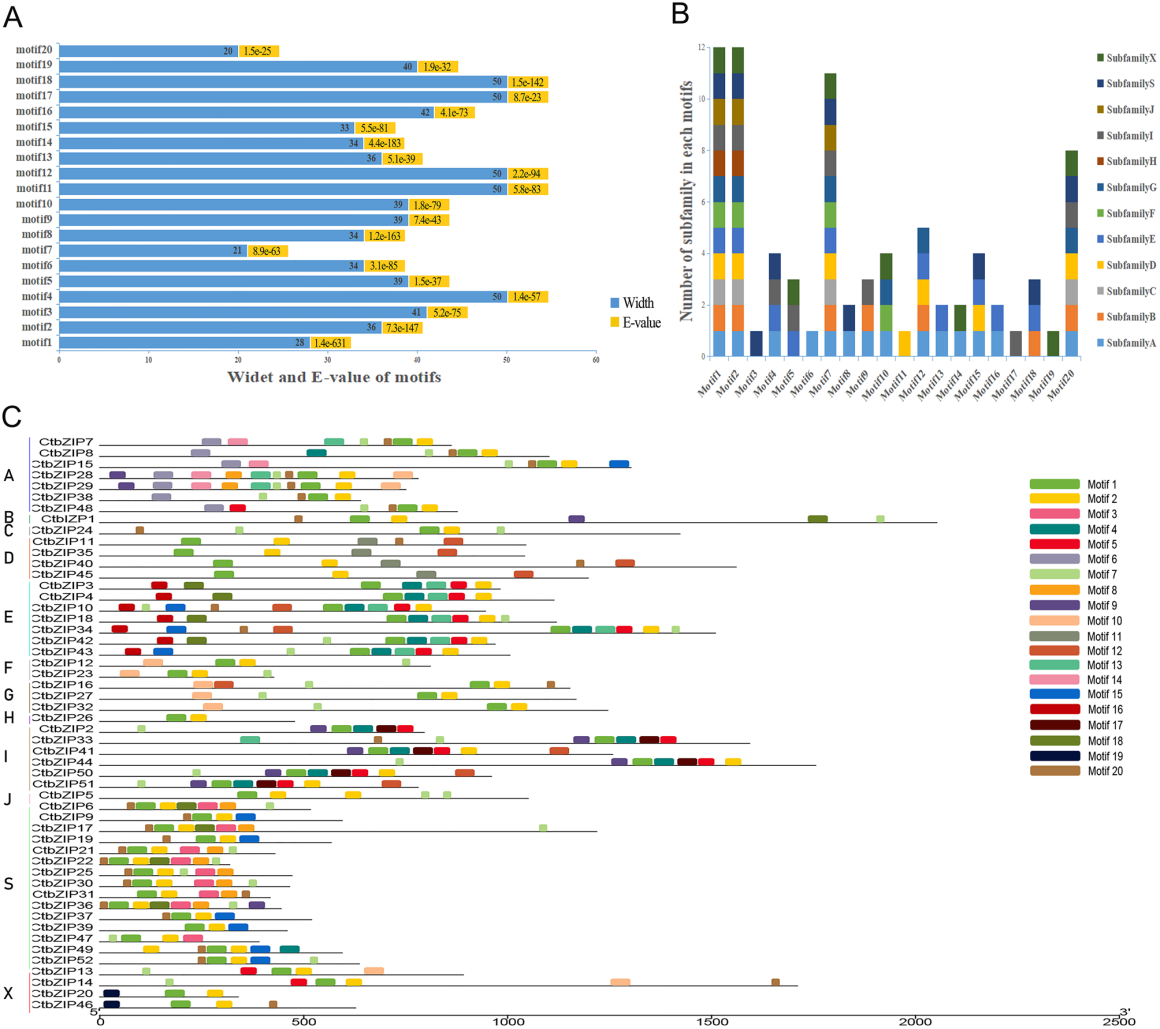
Conserved domain analysis of CtbZIP proteins in 12 subfamilies. (**A**) Width and E-value of sequence logos for 20 motifs. The blue portion indicates width and orange indicates E-value. (**B**) The number of subfamily in each motif. (**C**) Distribution of conserved structures in all 52 CtbZIP proteins.

Except the bZIP conserved domain, bZIP proteins usually contain other motifs which might bind potential functional sites thereby activating their function. Using ORF Finder tool of NCBI database, we found a complete open reading frame of all 52 CtbZIP transcripts. To find the conserved domains, Pfam database^28^ showed one or more of the intact conserved domains (bZIP_1, bZIP_2 and bZIP Maf while 20 conserved motifs were identified using MEME software^29^ the names and sequence logos of which are illustrated in Fig. 2. We counted the width and E value of each conservative motif using TB tools^30^ (Fig. 3A), and the distribution number of motifs in each subfamily was depicted (Fig. 3B). In terms of size, motif 20 was the shortest (20 aa) while motif 3, 11, 12, 17 and 18 were longest having 50 aa each. The motif average width lied around 38 aa. Interestingly, motif1 and motif2 were recognized as bZIP conserved domains and could be found in all of the subfamilies, however some subfamilies also had unique motif compositions (Fig. 3B,C). For example, subfamily A possesses a unique motif6, whereas motif11 is unique to subfamily D, motif 17 in subfamily I and motif13 in subfamily S. For the safflower specific subfamily X, CtbZIP20 and CtbZIP46 specifically contain motif 19, which are associated next to the N-terminus of the amino acid sequence and substantially identical to the bZIP conserved domain (Fig. 3C). All of these motifs indicate the group-specific functions for members in each group.

### Functional differentiation of CtbZIP TFs

**Figure 4:**
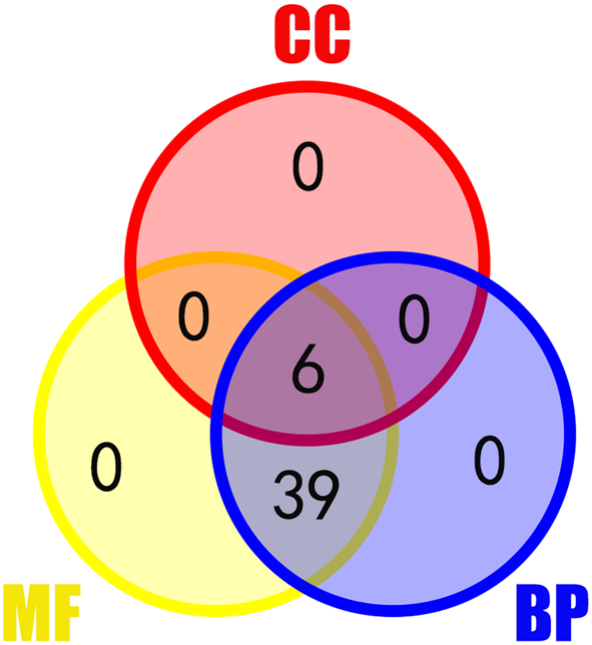
Venn diagram of the functional categorization of CtbZIP TFs. BP denotes biological process, MF stands for molecular function and CC for cellular component.

**Figure 5:**
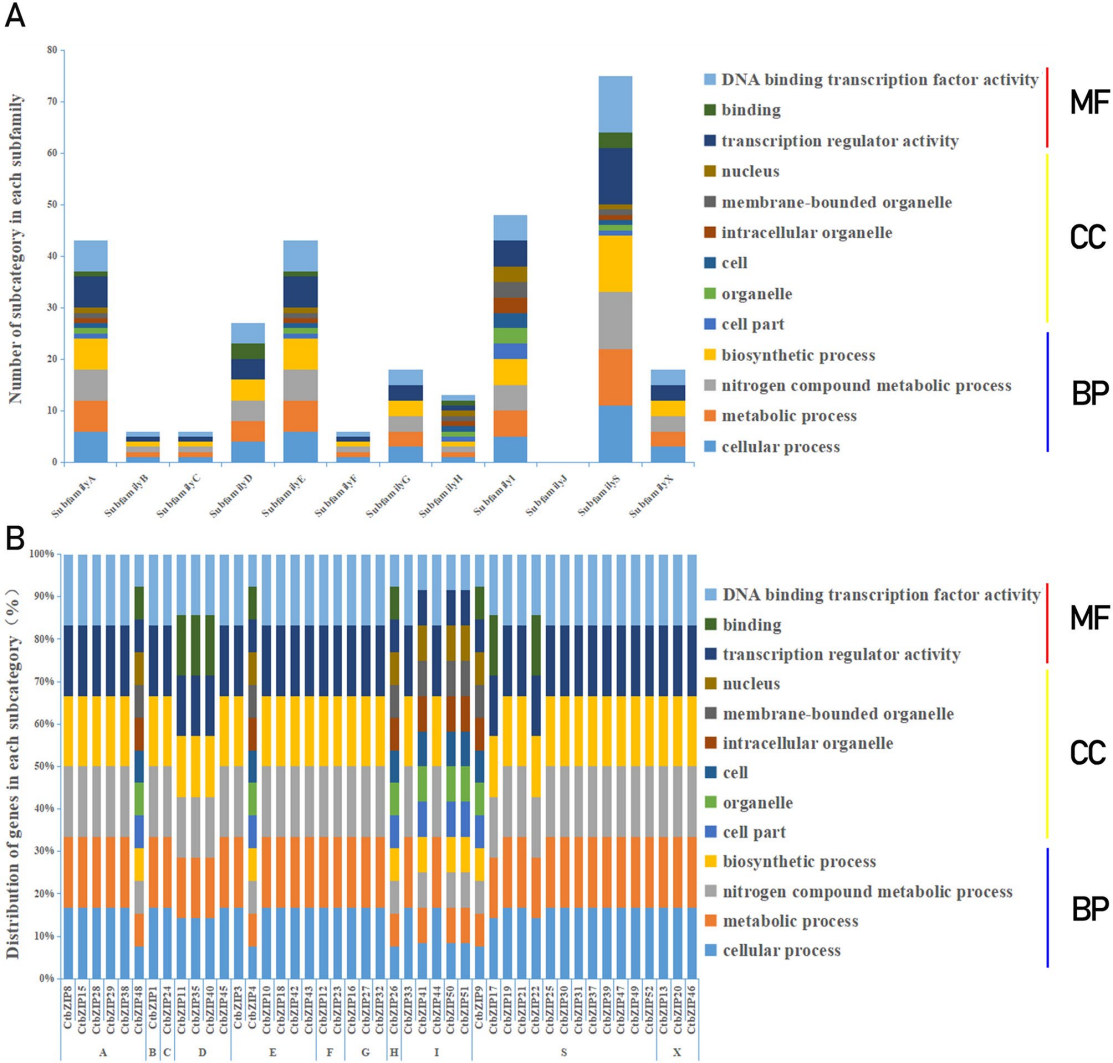
Variation of transcript function class in the CtbZIP family. (**A**) Distribution of each Subclass in 13 subspecies of Safflower CtbZIP TFs. (**B**) Variation of the functional categories of the 52 CtbZIP TFs.

Some motifs of bZIP TFs participate in a variety of physiological processes. To understand their function in the biological processes, we predicted the function of CtbZIP TFs in silico using Gene Ontology (GO) terms^31^. All of 52 CtbZIP TFs were analyzed, 45 of which categorized into three primary GO functional categories, biological processes (BP), molecular function (MF) and cellular components (CC) (Fig. 4). Among the 45 CtbZIPs, none was individually enriched into a certain GO functional category. Six CtbZIP TFs (13%) are enriched in three major categories CC, BP and MF while 39 CtbZIP TFs (87%) enriched in BP and MF. It can be seen that CtbZIP has many functions that affect the biological process of safflower. Besides, 45 CtbZIP TFs are classified into 13 subcategories, accounting for 57% of the enrichment data. The enrichment analysis showed that besides subfamily J, CtbZIP TFs of 11 subfamilies are enriched (Fig. 5A). At the same time, 6 subcategories are significantly enriched. The majority of CtbZIP TFs have DNA binding activity (Fig. 5B) and participate in the process of nitrogen metabolism. A number of CtbZIP TFs might respond to various abiotic stresses. All CtbZIP TFs have transcriptional regulatory activity, this allows them to regulate the growth and development of safflower. Based on these findings, the function of CtbZIPs may be associated with various biosynthetic and metabolic processes in response to abiotic and biotic stresses to affect the development of various tissues and organs.

### Expression profiles and network analysis of CtbZIP TFs

**Figure 6:**
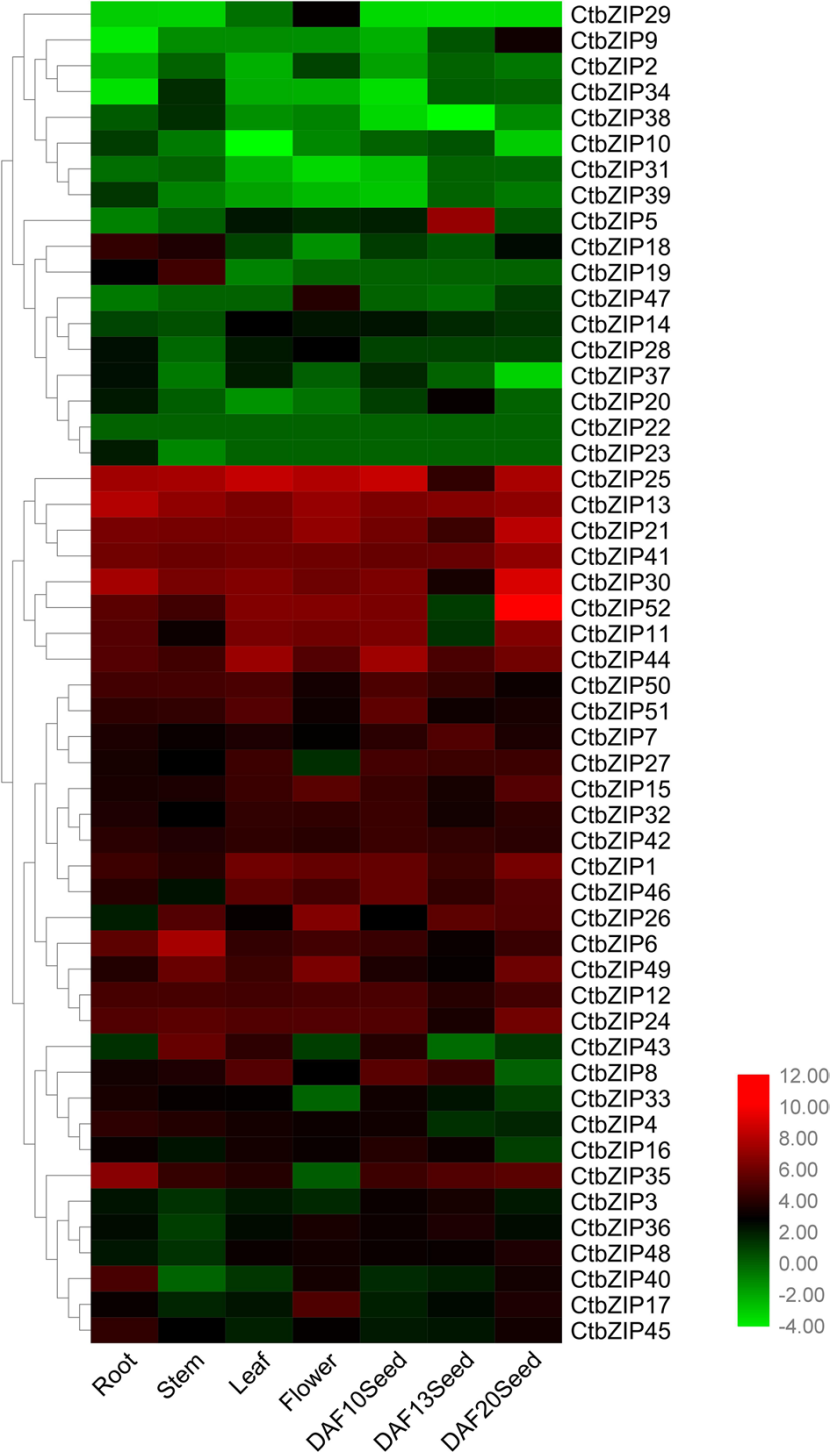
Heatmap of *CtbZIP* genes expressed among 7 tissues based on the fold change (log2) in RPKM values. The color scale at the top represents log2-transformed reads per kilobase million (RPKM) values.

The bZIP TFs are not only the most widely distributed and most conserved eukaryotic transcription factors, but their function is also diverse. The safflower bZIP TFs have a variety of functions and there are synergistic effects in the exercise of their functions. In order to explore the expression profiles and the interaction among the CtbZIP TFs, we analyzed their expression variation in different tissues, including roots, stems, leaves, flowers, DAF10-seeds, DAF13-seeds and DAF20-seeds by heatmap (Fig. 6). We noticed that *CtbZIP13* highly expresses in roots. *CtbZIP6* and *25* transcripts are abundant while that of *CtbZIP40, 23* and *29* are less in stem. *CtbZIP13* and *25* have higher expression in flowers than in other samples. High expression of *CtbZIP5* is observed in DAF13-seeds. Similarly, *CtbZIP52* highly expresses in DAF20-seeds. However, the expression levels of CtbZIP22 is almost the same in all of the 7 samples. The varied expression pattern indicates functional divergence of different groups of CtbZIP TFs. These results indicate that the functions of CtbZIP family are differentiated with differentiation in their expression.

**Figure 7:**
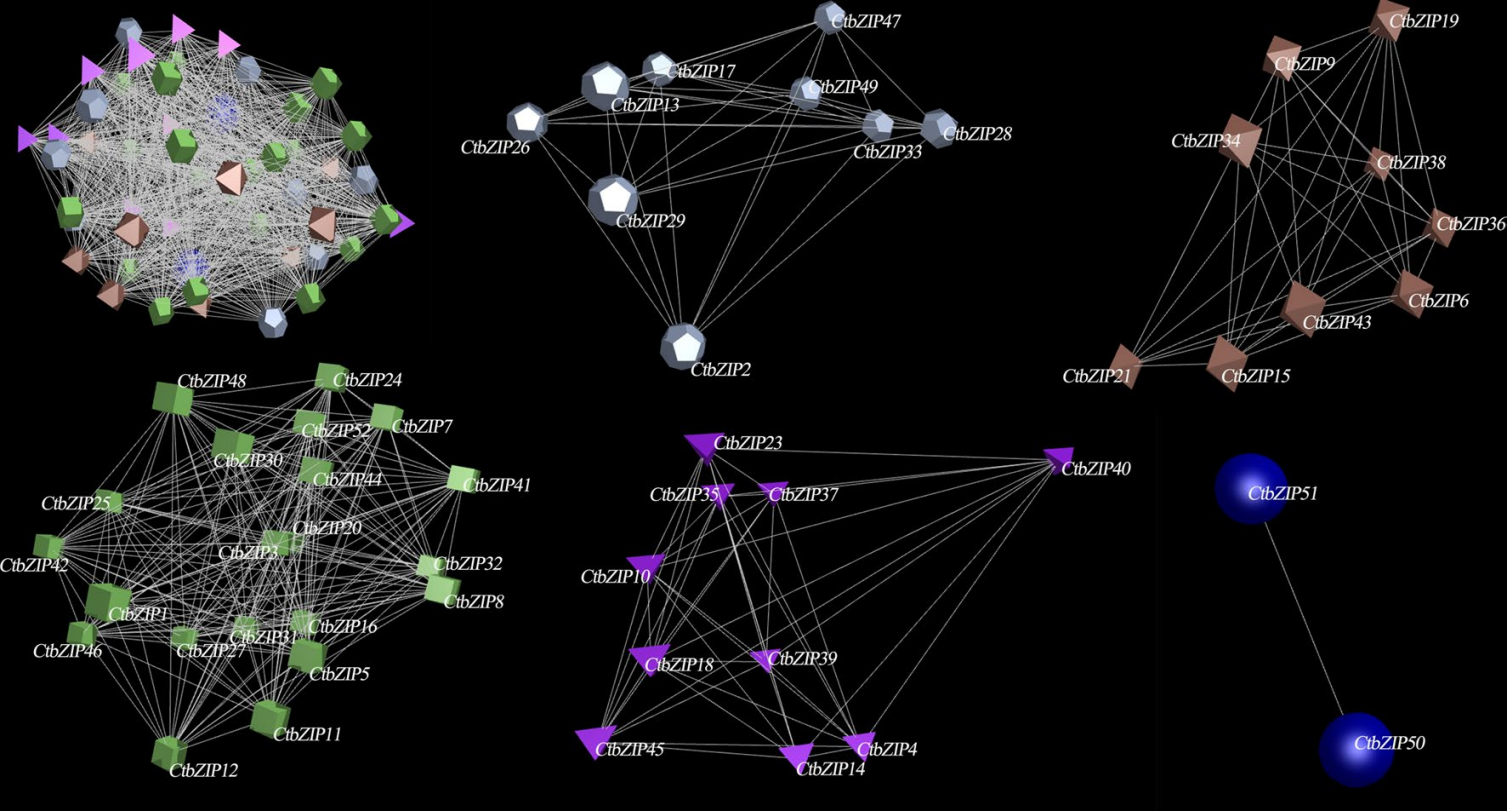
Network analysis of the CtbZIP TFs in seven different tissues of safflower. The co-expression network was constructed from the expression of 52 *CtbZIP* genes. The network was constructed at P ≤ 5.0 × e^−02^. It consists of 51 gene nodes and 1,199 edges. Five clusters in the network are shown separately.

We quantified the expressions of all 52 CtbZIP TFs in different tissues and seeds (of various developmental stages). The expression networks (p ≤ 0.05) (Fig. 7) were constructed using BioLayout Express 3D 3.2 software^32^. The CtbZIP TFs are a complex family with 51 nodes and 1,199 edges. Among them, 43 transcripts (85%) are more tended to have associated expression and form a co-expression network whereas the other 8 transcripts also exhibit weak co-expression. The network is composed of 5 clusters; the largest cluster contains sixteen transcripts, while the smallest cluster contains eight. There is a certain degree of related expression trend between these clusters and this tendency was statistically significant. These results indicate that although the functions and expressions of CtbZIP family members have dramatically diverged, they retain to some extent, the tendency of correlated expression and functional cooperation.

### Expression analysis of CtbZIP TFs in various tissues

**Figure 8:**
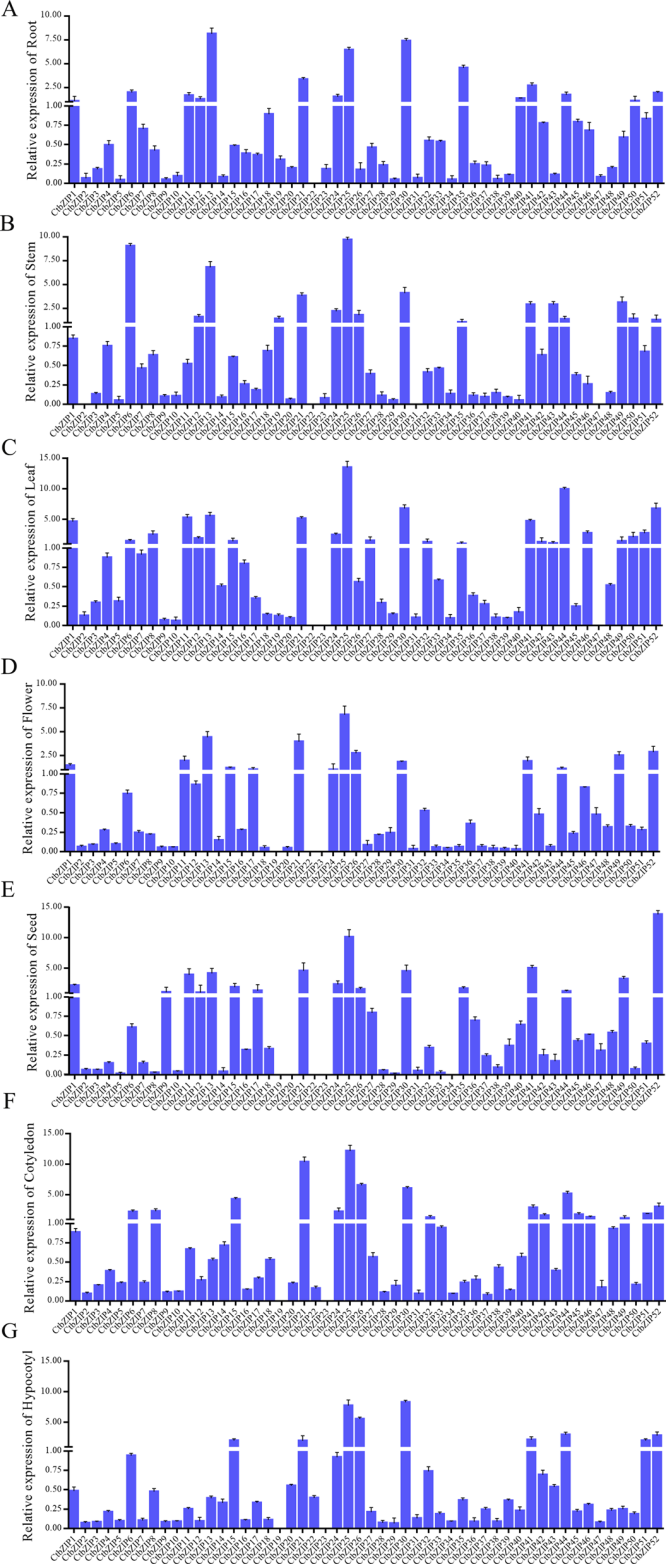
Relative expression profiles of the *CtbZIP* genes in various tissues. (**A**) Root, (**B**) stem, (**C**) leaf, (**D**) flower, (**E**) seed, (**F**) cotyledon, (**G**) hypocotyl. The reference gene used in RT-qPCR is EF1α. Values are average of three replicates ± SD. Asterisks indicate significant difference applying ANOVA (p < 0.05, p < 0.01 and p < 0.001).

To further verify the authenticity of the expression pattern, we detected the expression level of 52 *CtbZIP* genes in different tissues of safflower including roots, stems, leaves, flowers, seeds, cotyledons and hypocotyls using RT-qPCR (Fig. 8). The results showed that the *CtbZIP25* gene is highly expressed in all tissues and we speculated that it may be involved in various stages of plant growth and development. The *CtbZIP13* is highly expressed in root and might play a role in root growth. In seeds, *CtbZIP52* has the highest expression and might regulate the development of seeds. Likewise, *CtbZIP25*and *CtbZIP30* have higher expression in hypocotyls. The expression level of *CtbZIP6* and *CtbZIP25* peak in stem and they may affect the growth of the stem. Conversely, the expression level of *CtbZIP2, CtbZIP23, CtbZIP31* and *CtbZIP34* is relatively low in all tissues, among which *CtbZIP34* is the lowest in roots while *CtbZIP2, CtbZIP22, CtbZIP31* and *CtbZIP47* are the lowest in stems. Similarly, *CtbZIP23* and *CtbZIP47* are the lowest in leaves, *CtbZIP23* in flowers and *CtbZIP19, CtbZIP20* and *CtbZIP23* in seeds have the lowest expression. However, *CtbZIP22* gene expresses in cotyledon and hypocotyl after seed germination. This indicates that the *CtbZIP22* gene is specifically involved in seed germination. In short, the results of RT-qPCR show that the expression pattern of safflower is consistent with the predicted expression. According to this expression pattern, the function of CtbZIP TFs can be more effectively estimated.

### Expression level of CtbZIP TFs with GA3 treatment under different light radiation

**Figure 9:**
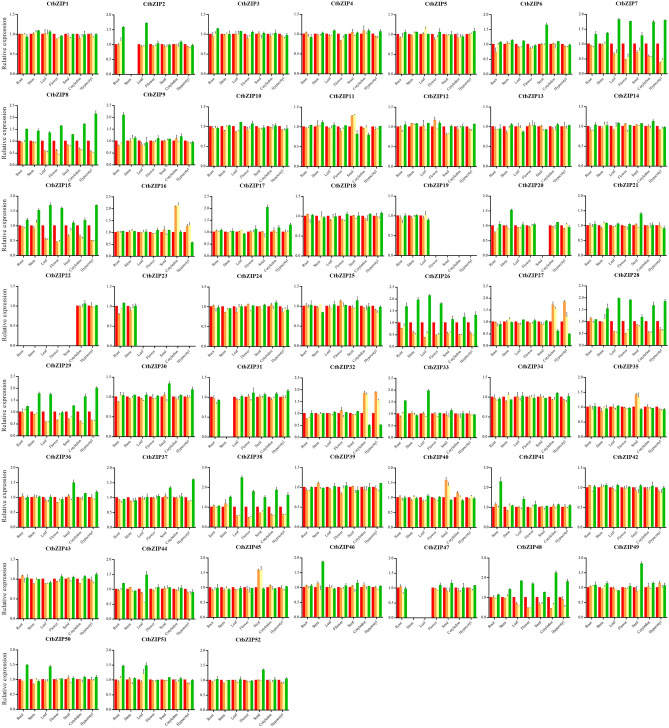
Expression profiles of *CtbZIP* genes after GA_3_ treatment under different light radiation. The red bars indicate no treatment under 16.8 MJ/m^2^ light radiation (group 1). The orange indicates no treatment under 5.04 MJ/m^2^ light radiation (group 2). Yellow indicates GA_3_ treatment under 5.04 MJ/m^2^ light radiation (group 3). Green indicates GA3 treatment under 16.8 MJ/m^2^ light radiation (group 4). The reference gene was *EF1α*. Values are average of three replicates ± SD. Asterisks indicate significant difference applying ANOVA (p < 0.05, p < 0.01 and p < 0.001).

In order to study the function of CtbZIP TFs, we detected the expression level of all 52 *CtbZIP* genes by RT-qPCR after GA3 treatment under different light radiation (no treatment under 16.8 MJ/m^2^ light radiation, no treatment under 5.04 MJ/m^2^ light radiation, GA3 treatment under 5.04 MJ/m^2^ light radiation and GA3 treatment under MJ/m^2^ light radiation) (Fig. 9). Among 52 genes, the expression of *CtbZIP15*, *CtbZIP26* and *CtbZIP28* highly increased in all the seven tissues, however, *CtbZIP28* and *CtbZIP38* upregulated in six tissues (excluding roots) after GA3 treatment under 16.8 MJ/m^2^ light radiation. Similarly, *CtbZIP2*, *CtbZIP33*, *CtbZIP50* and *CtbZIP51* in roots and leaves while *CtbZIP6*, *CtbZIP36, CtbZIP49* and *CtbZIP52* in seeds were up-regulated. *CtbZIP8* and *CtbZIP15* were significantly affected by illumination intensity and their expression increased in leaves, flowers, seeds, cotyledon and hypocotyl. Likewise, in seeds, *CtbZIP35*, *CtbZIP40* and *CtbZIP45* up-regulated after GA3 treatment under 5.04 MJ/m^2^ light radiation and *CtbZIP16*, *CtbZIP27* and *CtbZIP32* in cotyledon and hypocotyl were induced by GA3 and light.

## Discussion

Safflower is an important plant used for ornamental, food, feed and medicinal purposes. In terms of tolerance for abiotic stresses such as water deficit, it is a tough plant however, for increasing demand of edible oil as well its vast pharmaceutical properties, its improvement seeks comprehensive understanding through omics. Omics by combining genomics, transcriptomics, proteomics and metabolomics (as solving a puzzle) attempts to obtain a clear picture of molecular and biochemical circuitries underlying primary and secondary metabolites/products^33^. In the same race, we tried to unravel an important class of transcription factors in *Carthamus tinctorius* L. Transcription factors express genes thus are essentially present in all organisms. They comprise several classes holding fundamental role in various growth and developmental processes. The *bZIP* gene family plays role in plant growth and seed and fruit development^34^. Plant bZIP TFs preferentially bind promoters containing ACGT core sequence including A- (TACGTA), C- (GACGTC) and G- (CAGTG) boxes^35^, however nonpalindromic targets have also been reported^36,37^. The genome-wide analysis of CtbZIPs would aid in their further functional analyses as well as safflower breeding research.

Our genomic survey identified 52 members in *Carthamus tinctorius* bZIP TF family. These TFs constitute a large families in all organisms reported to date. *CtbZIPs* also look a big gene family however, as compared to Arabidopsis (78 TFs), rice (89), maize (125), *Brassica napus* (247) and soybean (131), safflower got a relatively small bZIP family. Based on phylogenetic reconstruction (Fig. 1), we categorized CtbZIPs into 13 subfamilies (A-J, S and X) according to their relevance in Arabidopsis^4^, rice^5^, *Ricinus communis*^26^ and *Camellia sinensis*^27^. This categorization was further supported protein structure analyses. None of CtbZIP proteins clustered into subfamily K and M indicating loss of these proteins throughout safflower evolution.

CtbZIPs protein structure analyses revealed 20 motifs in total, same as reported in *Manihot esculanta*^38^, which were named sequentially from motif1 to motif20 (Figs. 2, 3). Relating their motifs to some known motifs revealed some functions of CtbZIP TFs. The motif2 was further identified as the extension of the leu zipper region, closely related to motif1. The motif4 was a new highly conserved cysteine-rich sequence which might be involved in protein–protein or protein-DNA interactions. In most of the cases, motif1 and motif2 conserved domains are located next to each other, however, some motifs are located far from each other. The maximum distance between two motifs is found in CtbZIP45 of subfamily D. In addition, there are three motifs (motif4, 5, 13) between bZIP domains in subfamily E of CtbZIP TFs, and motif1 and motif2 together with three motifs form a conserved structural group, as the subfamily E of OsbZIPs^5^. The same situation exists in the subfamily I of CtbZIP TFs, motif1 and motif2 together with motif4, 5, 9, 17 form a conserved structural group, but motif9 is not between motif1 and motif2. The conserved groups of E and I subfamilies exist near the C-terminus which predicts that the functions of subfamily E and subfamily I could make a significant difference with other subfamilies. CtbZIP26 only contains the bZIP domain (motif1 and motif2) in the subfamily H, which confirms that the function of CtbZIP26 is more conservative. The motif11 in subfamily D is a conserved structure of Dog1 (PF14144) also found in *Arabidopsis* bZIP^4^. This family appears to be a highly specific controller of seed dormancy. On one hand, MEME results further prove that outcomes of Hidden Markov Model (HMM) have high reliability. On the other hand, they also reveal the functional diversity of CtbZIP family. These analyses are an important starting point for further functional verification.

The genome-wide expression prediction of *CtbZIPs* genes flaunted their differential transcript level in various developmental stages and tissues. As shown in Fig. 6, there seems a vast level of divergence in expression pattern with respect to tissue type and seed stage. The varied expression pattern indicates functional divergence of different groups of CtbZIP TFs, which predicts that the functions of CtbZIP family vary with variation in their expression. We quantified the expressions of all 52 CtbZIP TFs in different tissues and seeds (of various developmental stages). The network is composed of 5 clusters as shown in Fig. 7. There is a certain degree of related expression trend between these clusters and this tendency was statistically significant. These results indicate that although the functions and expressions of CtbZIP family members have dramatically diverged, they retain to some extent, the tendency of correlated expression and functional cooperation. To verify the transcript abundance of *CtbZIPs* genes, we used RT-qPCR and evaluated their expression in root, stem, leaf, flower, seed, cotyledon and hypocotyl (Fig. 8). The results of RT-qPCR showed that the expression pattern of safflower is consistent with the predicted expression. According to this expression pattern, the function of CtbZIP TFs can be more effectively estimated.

In the process of plant growth and development, light and hormone are the key factors that directly affect these two processes. At present, it has been confirmed that the A subfamily bZIP members of *Arabidopsis thal*iana are mainly involved in ABA signaling^39^ whereas H and G subfamilies regulate photoresponse^14,40^. In rice, *OsbZIP12* has been reported as a positive regulator of ABA signalling^41^ while in *Medicago esculenta*, *bZIP11, 27, 52* and *64* were upregulated at time points of ABA treatment^38^. In *Ipomoea trifida*, eight *bZIP* genes were upregulated at least in one tissue type as well as one time point, in response to ABA treatment^42^. AtbZIP16 has been reported to regulate early development of seedling by integrating hormone and light signalling pathways thereby promoting germination as well elongation of hypocotyl^43^. Under RL (Red Light) treatment, *ClabZIP6* and *ClabZIP56* were significantly induced while *ClabZIP37* and *ClabZIP22* were repressed in leaves of *Citrullus lanatus*^44^. Figure 9 depicts that changes in expression of CtbZIPs under GA3 and light reveal that some of *CtbZIP* genes might be directly or indirectly affected by light intensity and hormones. These results provide a basis for further exploration of the function of CtbZIP TFs.

In summary, our study provides genome-wide analysis of the safflower *bZIP* family. We accurately screened 52 CtbZIP TFs, and divided them into 12 subclasses by identifying the conserved homology between them. Their basic physical and chemical properties were analyzed including ORF, number of amino acids and conserved structural positions. A total of 20 conserved structures are found in CtbZIP TFs family. All CtbZIP TFs contain a typical conserved bZIP_1 domain. For the enrichment analysis of the CtbZIP TFs, we found that 45 of the 52 CtbZIPs were enriched, and among the 45, none of the genes were individually enriched into a certain GO functional category. Six CtbZIP TFs were enriched in three major categories CC, BP and MF, and 39 CtbZIP TFs are enriched in BP and MF. A total of four clusters within the CtbZIP TFs were discovered, which constitute a complex interplay network. The expression patterns of the CtbZIP family were predicted and verified by heat map and qRT-PCR. This study improves our understanding of safflower *bZIP* TFs and lays the foundation of cultivating new cultivars of safflower through molecular breeding methods.

## Methods

### Plant materials and treatments

The JiHong No. 1 safflower seeds purchased from safflower edge Co. Ltd. in Xinjiang of China, were cultivated in experimental field of Jilin Agricultural University for multiplication. The collected seeds of safflower were germinated in soil and allowed to grow at 23 ± 2 °C in growth room. It takes about 7 days to sprout cotyledons and hypocotyls, flowers in approximately 100 days while seeds in about 135 days. For light treatment, some safflower plants were grown under normal light radiation (16.8 MJ/m^2^) while another set of plants under weak light radiation (5.04 MJ/m^2^). For GA3 treatment, the plants that grew after flowering were sprayed with 50 mg/L GA3 once daily for 5 days. Each experimental group was sprayed simultaneously at 10 am. We collected various tissues, such as leaf, stem, root, flower, cotyledon, hypocotyl and seeds, immediately froze in liquid nitrogen and stored at − 80 °C for further use.

### Identification and characterization of CtbZIP TFs

The sequences of *CtbZIP* were obtained from the safflower genome database (Accession: PRJNA399628 ID: 399628). We downloaded HMM profile of bZIP_1 (PF00170) from Pfam database^28^ (https://pfam.xfam.org/) and the similar sequence of bZIP_1 was searched using Hidden Markov Model (HMM) as the query (P < 0.001). To avoid missing possible *bZIP* members, NCBI BLAST was performed using the known *Arabidopsis bZIP* sequences (downloaded from the TAIR, https://www.arabidopsis.org/), as queries against the safflower genome database^26^. All of the possible bZIP TFs were screened according to the significant e-value < 1 × 10^–5^ in our data. In addition, the conserved bZIP domains were predicted using SMART^45^ (https://smart.embl-heidelberg.de/) and Search Pfam^28^ (https://pfam.xfam.org/search/sequence) in all of the possible bZIP TFs. Therefore, the high-confidence bZIP TFs were screened, which were named as CtbZIP. Afterwards, we analyzed the physical and chemical properties of the predicted high-confidence CtbZIP TFs by ProtParam online tool^46^ (https://www.expasy.org/).

### Phylogenetic analysis of the CtbZIP proteins

The bZIP protein sequences of *Arabidopsis* and *Ricinus communis* were downloaded from database of PlantTFDB (https://planttfdb.cbi.pku.edu.cn) and that of rice were downloaded from the Rice Genome Annotation Project^47^ (https://rice.plantbiology.msu.edu/index.shtml). Multiple alignment of the full-length bZIP sequences of safflower, *Arabidopsis*, rice and *Ricinus communis* was executed using Clustal X 2.0 program^48^ and saved in the Clustal X file format. Using MEGA 7.0 program^49^, we constructed a cladogram tree with 1,000 bootstrap replications and Neighbor-joining algorithm. The phylogenetic tree was modified using the iTOL online software^50^ (https://itol.embl.de/login.cgi).

### Motifs analysis of CtbZIP proteins

We searched the open reading frames of *CtbZIP* genes through the ORF finder at NCBI (https://www.ncbi.nlm.nih.gov/gorf/gorf.html). CtbZIP transcripts were analyzed in the Pfam^28^ (https://pfam.sanger.ac.uk/) protein database. Analysis of the conserved motifs in safflower CtbZIP TFs were further carried out by multiple EM for motif elicitation software (MEME^29^) (https://meme.sdsc.edu/meme/cgi-bin/meme.cgi) with default parameters. The maximum number of motifs was set to 20 and motif width to 6-50aa. Whereafter a conservative structure was generated using TBtools^30^ (https://www.tbtools.com/). The related motif information used is listed in Table S2.

### Gene ontology annotations of CtbZIP TFs

The functions of the CtbZIP TFs were categorized in silico using Blast2GO software^31^ (https://www.blast2go.com/). The GO functional categorization of 52 CtbZIP TFs was used into each subcategory for enrichment analysis. The enrichment of the number of CtbZIP transcripts categorized into each subcategory was determined by Chi-square test.

### Network analysis of the CtbZIP TFs

The construction of the co-expression network is conceptually simple and intuitive. Through the similarity of gene expression, the possible interactions of gene products can be analyzed to understand the intergenic interaction. The various traits are the result of genetic interactions. In order to excavate the network of interactions during *CtbZIP* genes family, we used the R programming language and software^51^ to calculate Pearson correlation coefficient. A gene co-expression network was constructed using BioLayout Express 3D Version 3.2 software^32^.

### Gene expression patterns analysis

To investigate the *CtbZIP* gene family expression patterns, the high-throughput safflower transcriptome sequencing data were used to analyze the *CtbZIP* gene expression patterns in various tissues for roots, stems, leaves, flowers and DAF10, 13 and 20 seeds. The expression estimations of *CtbZIP* genes were normalized and represented in the form of RPKM (reads per kilo base per million mapped reads), and fold change (log2) values were calculated through the ratio of gene expression to draw heatmaps with R^51^ and TBtools^30^ software.

### RNA extraction and cDNA synthesis

The experimental materials (various tissues: root, stem, leaf, flower, seed, cotyledon, hypocotyl) were pulverized adequately and put into centrifuge tubes. Total RNA of various tissues was isolated using Trizol (Invitrogen, Carlsbad, CA, USA), according to the instructions of the manufacturer. The extracted total RNA was treated with RNase-free DNase (Promega, USA) to remove the genomic DNA contamination. RNA quality was checked on OD260/280 values by Nano Drop 2000 (ThermoFisher Scientific, Beijing, China) and 1.2% agarose gel electrophoresis. The cDNA was synthesized from total RNA isolated from various tissues using the PrimeScript RT reagent kit with gDNA Eraser (Takara, Japan), according to the manufacturer’s protocols. First, 2 μL 5 × DNA Eraser buffer, 1 μL gDNA Eraser, 2 μL total RNA (about 1,000 ng) and 5 μL RNase free ddH_2_O were mixed in tube and incubated at 42 °C for 2 min to remove DNA. The purified RNA was reverse-transcribed into cDNA by adding 4 μL 5 × PrimeScript buffer, 1 μL PrimeScript enzyme mix I, 1 μL RT primer mix and 4 μL RNase free ddH_2_O into the above-mentioned reaction and incubated at 37 °C for 15 min followed by 85 °C for 15 s. The cDNA was stored at − 20 °C.

### Real-time fluorogenic quantitative PCR

Real-time fluorogenic quantitative PCR (RT-qPCR) was performed using SYBR Premix Ex Taq II kit (Takara, Japan) and Stratagene Mx3000P thermocycler (Agilent) to monitor DNA products. The most stable housekeeping reference gene (*EF1α*) was selected for the expression analysis in various tissues. The relative expression of *CtbZIP* was normalized to the expression of *EF1α* and expressed relative to the level in various treatment. Gene-specific primers designed for the *CtbZIP* genes are listed in Table S3. RT-qPCR amplification was performed in 15 μL reaction volume containing 500 ng template cDNA (1 μL), 0.3 μL primer (10 m), 7.5 μL SYBR Premix Ex Taq (2×), 0.3 μL ROX Reference Dye (10 m), and 5.6 μL DEPC ddH_2_O. RT-qPCR profile was set as an initial denaturation at 95 °C for 5 min, followed by 40 cycles of 95 °C for 5 s and annealing at 60 °C for 30 s. The fold change in relative expression level was calculated using $2^{{ - \Delta \Delta {\text{CT}}}}$ method.

### Statistical analysis

The experiment was designated for three random replications. All data were analyzed by one-way analysis of variance (ANOVA) and all means were separated at the P < 0.05 level. The different tissues and GA3 treatment by the biological significance of the differential expression were analyzed.

## Supplementary information

Supplementary Information.

## Abbreviations

BP: Biological process
bZIP: Basic region/leucine zipper
Ct: *Carthamus tinctorius* L.
CC: Cellular component
GRAVY: Grand average of hydropathicity
HMM: Hidden Markov model
Kda: Kilo Dalton
MF: Molecular function
MW: Molecular weight
RPKM: Reads per kilobase per million mapped reads
TFs: Transcription factors
