# Supplementary material for: Genome-wide identification and expression analysis of bZIP gene family in Carthamus tinctorius L
Source: Sci Rep. 2020 Sep 23;10:15521. doi: 10.1038/s41598-020-72390-z (PMC7511407; doi:10.1038/s41598-020-72390-z)
Supplement: Supplementary file 1 — Supplementary Information. [file 41598_2020_72390_MOESM1_ESM.pdf]

Genome-wide identification and expression analysis of *bZIP* gene family in *Carthamus tinctorius* L.

Haoyang Li[1#], Lixia Li[1], Guodong ShangGuan[1], Chang Jia[1], Sinan Deng[1], Muhammad Noman[1], Yilin Liu[1], Yongxin Guo[1], Long Han[1], Xiaomei Zhang[1], Yuanyuan Dong[1], Naveed Ahmad[1], Linna Du[1]*, Haiyan Li[1]*, Jing Yang[1]*

[1.]College of Life Science, Engineering Research Center of the Chinese Ministry of Education for Bioreactor and Pharmaceutical Development, Jilin Agricultural University, Changchun130118,China;

*Correspondence:
dulinna0918@163.com ; Tel.: +86-0431-84533428 (LN.D)
hyli99@163.com; Tel.: +86-0431-84533428 (HY.L);
yangjing5122010@163.com; Tel.: +86-0431-84533347 (J.Y);

Table S1: Information regarding the molecular weights (MW) in Daltons (Da), isoelectric points (PI) and GRAVY (Grand Average of Hydropathicity) of the 52 identified CtbZIPs

S. No.	Name	MW(Da)	PI	GRAVY
1	CtbZIP1	73869.99	6.74	-0.483
2	CtbZIP2	26462.28	4.86	-0.753
3	CtbZIP3	36455.4	8.46	-0.565
4	CtbZIP4	41652.83	7.83	-0.981
5	CtbZIP5	86187.57	5.04	-0.918
6	CtbZIP6	18683.8	5.04	-0.452
7	CtbZIP7	31883.23	8.98	-0.752
8	CtbZIP8	40599.66	9.05	-0.783
9	CtbZIP9	22638.36	7.16	-0.849
10	CtbZIP10	35103.86	5.9	-0.88
11	CtbZIP11	85532.14	5.06	-0.853
12	CtbZIP12	29617.17	5.89	-0.757
13	CtbZIP13	32369.78	6.07	-0.75
14	CtbZIP14	65003.69	8.73	-0.798
15	CtbZIP15	46115.72	9.78	-0.645
16	CtbZIP16	40785.79	6.28	-0.925
17	CtbZIP17	44410.39	5.51	-0.635
18	CtbZIP18	41812.1	7.15	-0.965
19	CtbZIP19	21976.2	5.37	-0.983
20	CtbZIP20	13139.16	9.25	-0.596
21	CtbZIP21	15899.11	8.89	-0.589
22	CtbZIP22	11980.6	9.73	-0.632
23	CtbZIP23	16020.97	8.36	-0.869
24	CtbZIP24	50338.58	6.04	-0.759
25	CtbZIP25	17741.79	5.69	-0.715
26	CtbZIP26	32551.57	6.8	-0.689
27	CtbZIP27	40776.35	7.75	-0.685
28	CtbZIP28	28469.83	5.24	-0.648
29	CtbZIP29	27181.37	5.97	-0.437
30	CtbZIP30	17493.48	6.14	-0.799
31	CtbZIP31	16048.17	9.52	-0.766
32	CtbZIP32	44040.17	6.04	-0.984
33	CtbZIP33	58204.11	6.49	-0.643
34	CtbZIP34	56271.68	6.42	-0.785
35	CtbZIP35	39375.83	6.17	-0.374
36	CtbZIP36	16197.41	8.88	-0.483
37	CtbZIP37	20320.73	6.16	-0.831

38	CtbZIP38	22953.88	9.2	-0.482
39	CtbZIP39	17671.69	6.52	-0.889
40	CtbZIP40	57703.56	6.58	-0.593
41	CtbZIP41	46132.98	6.02	-0.866
42	CtbZIP42	17432.6	5.29	-0.23
43	CtbZIP43	36904.57	5.43	-0.902
44	CtbZIP44	63901.99	6.64	-0.976
45	CtbZIP45	44399.14	5.56	-0.392
46	CtbZIP46	22592.17	6.08	-0.653
47	CtbZIP47	15250.49	9.18	-0.932
48	CtbZIP48	32735.02	8.81	-0.88
49	CtbZIP49	23079.63	5.45	-0.917
50	CtbZIP50	35465.92	5.5	-0.861
51	CtbZIP51	28823.96	9.08	-0.935
52	CtbZIP52	24475.93	5.4	-1.008

Figure S1. Phylogenetic reconstruction of Arabidopsis and safflower bZIP proteins.

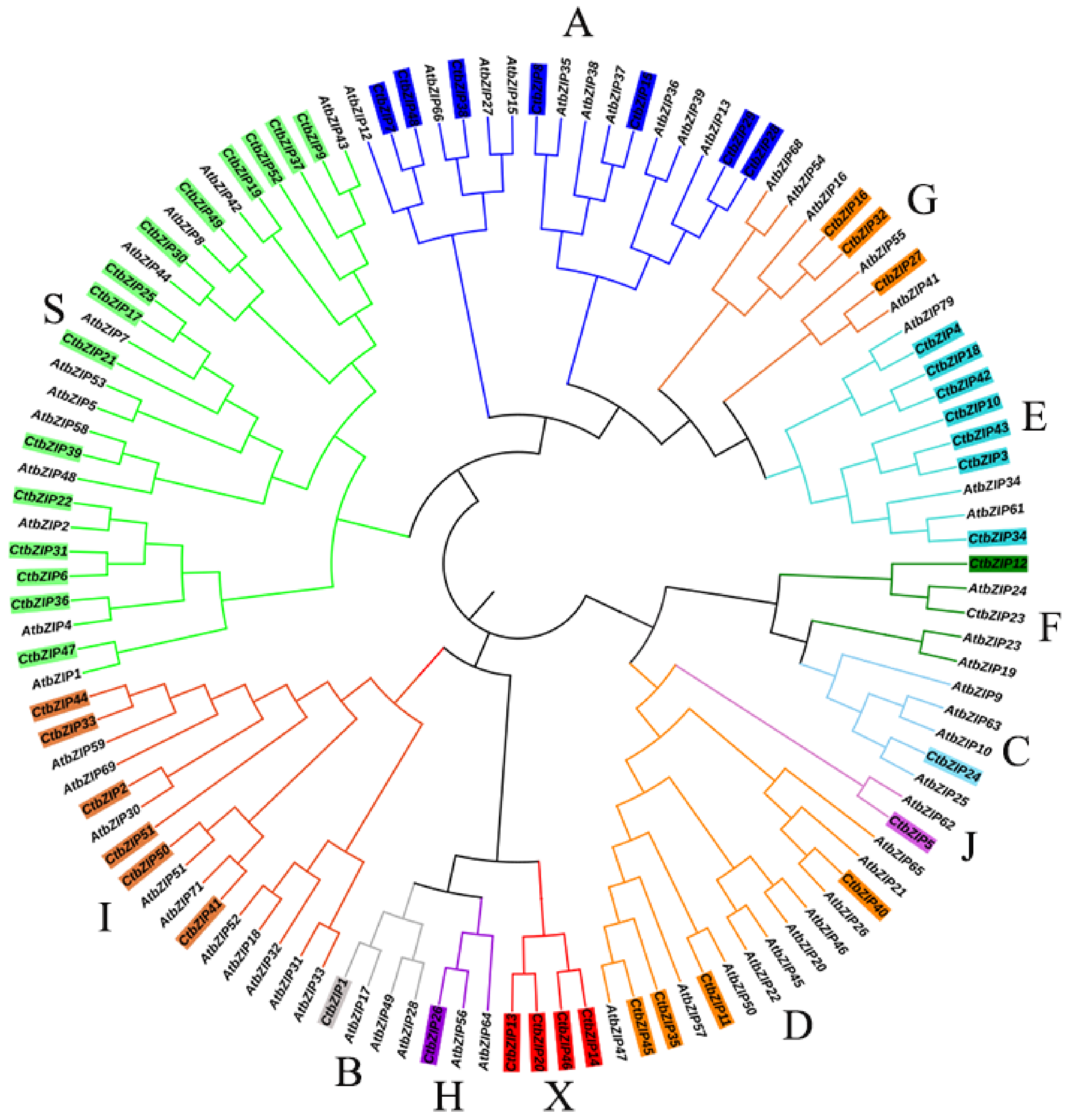

Table S2. CtbZIP TFs conserved structure sequence and information

Serial number	Predicted conserved structural sequence	width	E-value
Motif1	[RKQTML][RKET][QRAVKLI][KRDATG][RTGA][MILK][ILQAV][SAKQR][NKP][RQ][EQ0[SAF][AV][RAQ][RKQ][SYTA][RK]K[QKLMA][AQRK][YHDER][LIVTA][DAQT][EDQ]L[EAVTM]	28	1.4E-631
Motif2	[LVMF][EQDNV][RAQHE][QED][NSRT][SLIAN][VIGY][LI][SRTNK][ASVML][QEDR][NVLIA][SKATN][EAV]L[KSTG][QHLFR][RQ][LIF][QDAE][SANYTR][LM][NEA][QE][IQEDM][IKAQV][QLANI]LFIV][KMRI][DNY][ALQC][LQHPE][NSQHPA][ETIQ][AYD][LYCT]	36	7.30E-147
Motif3	[TSQL][QRKE][VLAI][ELSVMIF][WQASRYV]L[RTKSQ][NEVT][EDQK][NYK][HSQLNEA][TSGQLE][LMI][VKMLI][DASK][KERQ][LIVM][NTSQE][RHSN][FLIA][TSRYLA][EQVKD][TSNE][HYSFWR][EKDAVN][QRKN][VLATMI][VLKA][ERLAY][EQNSD]N[ADRTNM][VQKSRL][LM][KRLI][EKSAG][ERQKD][LVT][STKGEVML][ETNKD][LTSMIEA]	41	5.20E-75
Motif4	TNLNIGMDYWSG[TA]N[TS][SP]NIPAMRG[KH]VTSAPVAGGMV[TA]AGSR[ED]SM[QP][SP]QLWLQ	50	1.40E-57
Motif5	[PD]P[TSN]W[VM]D[DE]F[LV]DFSS[AT][RK]R[SG]SHRRS[VT]SD[PS][IA]AF[IL]E[TIA]P[SPM][FL][IG][NED][ED][CS]	39	1.50E-37
Motif6	[GFW][SRL][GDH][DSN][LIS][SLA][RG][MTID][PWG][DE][NPK][PLG][PF][KP][YRQHC][KL][TSRGA]HRR[AS][NLHA]S[DE][IS][PLIG][TF][LGV][PFL]DS][TDG][IL][SLA][QFE]	34	3.10E-85
Motif7	[WMG][GP][PG][YPL][QGP][THV][MPA][PMY][PH]P[YH][VAP]A[MI][YA]P[HM]G[GY][VIP][VIW]	21	8.90E-63
Motif8	[KHND][TSP][NFGE][SM][LV][EDTI][SF][GS][SNQA][LGE][EFM][TFM][SET][SG][IAVS][ED][AL]KK[SIA][MI][AS][PNDA][DAE][KN]LAE[LI][AW][LT][VT]DP	34	1.20E-163
Motif9	[QG][SG][ST][LI][VI][NSA]LT[LF][DN]E[VL]Q[TQNH][TQ][LM]G[DSG][LNI]GK[PD][LF][GST]SMN[ML]DEL[LI]K[SN][VI]W[TNI]AE	39	7.40E-43

Motif10	[RK][AVL][EV][AVSD]L[TKRA][EWSNI]EN[LSQEA][TSLK]L[KR][SAQK][ES][VILK][SNQE][RT][LIA][TRYS][SVKED][NEKD][YSW][DEA][QKSN][LI][KVRL][LAYT][LI][KVRL][LAYT][QSKE]N[AIE][SKLC][LM][KLI][EGA][RKQE][LVQM][KGSI][KENC]	39	1.80E-79
Motif11	[RH][IV][LIF]VD[SD][TSGF][LVM][NA]HY[YHDA][END][LI]F[QTR][MLI]K[AG][MVD]AAK[SA]DV[FC][YH]L[MI][TSNH]G[LVM]W[RTK][TS][PS][VTA]ER[FLC]F[QL]W[IML]GG[FP]RP	50	5.80E-83
Motif12	F[LV][NR]QAD[HN]LRQQ[TV]L[QH]Q[ML][YSH]RILT[VTI][RH]QAA[RK][GCA][LF][LI][VA][VI][GS]EY[FY][QNG]RLR[AV]L[SN]SLW[AS][AS]R[PF]	50	2.20E-94
Motif13	[QK][LVSI][VFH][DSNG][LVI][PYTSC][STN][LR][SRQI][QEH][SVA][CS][YQKM][QC][AE]E[QED][APS]L[TS][QSL]G[MHL][ESD][KLQ]L[QAH][QK][TCS][LS][ANV][QNED][YTSGA][IAV][TSA][SIC]	36	5.10E-39
Motif14	Y[NS]GK[HN][SY][LS]LPPKSPFPS[IV]AP[SF]Y[AS]DY[VIA][SP][TSN][AS]A[IS]GPKG	34	1.40E-183
Motif15	PDW[TA]GFQ[PA]Y[SP]PMPPHG[YF]LASSPQ[PA]HPYMWGVQH	33	5.50E-81
Motif16	[QM][PD][TSN]W[VM]D[DE]F[LV]DFSS[AT][RK]R[SG]SHRRS[VT]SD[PS][IA]AF[IL]E[TIA]P[SPM][FL][IG][NED][ED][CS][RS][NGC]	42	4.10E-73
Motif17	E[YRH][AR][AV][QK][VHD]E[YQ][RW][LM]RN[LM][TNI]SA[PL]SW[GA]SQDFD[LIF]Y[KT]D[VSA][RQ]N[PMA][SP]FYAE[PH]N[RPA][MI][QMI][RNK]N[KQ][TNA][RW][TDA][WS]	50	8.70E-23
Motif18	KIPPPPLPSERLSGGFSFDNPIHOONVDGVVGFGIGVEEMGGRGKRRAI	50	1.50E-142
Motif19	[VN][TVI][NSP][ST]G[IA]A[ATM]F[EKD]M[EA]Y[ESD][RQML]W[VL][AED][ED][QD][QRN][KR][KLH][DTM][NDA][EA]L[RK][KTD][VMGA]L[QMH][TS][PHD][LVM]][SP][DE][TLIG][EQD]L	40	1.90E-32
Motif20	S[LP][TSPD][LP][SPA][RTS][TD][LI][STR][QKT]KT[VM][DE]E[VL]W[RQK][DGE][IL]	20	1.50E-25

Table S3. The qRT-PCR primers of CtbZIP family genes

Gene	forward primer	reverse primer
CtbZIP1	GAGGAATCCGTTAGGGTTTTG	CAGACTCAGGCGAAGGACA
CtbZIP2	GAGAAATCTGGTGGTGAATGTG	CTAATATCGTCGGGTAGAGTGA
CtbZIP3	CGGAGCAAGGAGAAAGTAACAAT	TTGTGGAAAGCCCGAAAC
CtbZIP4	TGATGCGTCTAATACCAAGGC	CCTCTGAACCTTCTGCCTGT
CtbZIP5	AATGCTTGAAGGCACAGATG	AGTGGCAGAAAGGGAGGTT
CtbZIP6	GAACAGGGAATCGGCGA	CGCTGATGCTGGAGACG
CtbZIP7	AGGGGTGGTTGCGGAAT	ATAACCCATCTCCATCACCG
CtbZIP8	AGAACGCTTAGCCAGAAA	CCTCTAATGTCATCTCACCC
CtbZIP9	AGGAGGATGATATCGAACCG	CGAAAGCGTAGGAGGATGATAT
CtbZIP10	CATATCTGGACCATCAACGAC	CTATCTCTGCCTTCAATGCTTC
CtbZIP11	CTTTCAGTGGCTTGGAGGAC	GCATCCTCGGCTTGCTTAC
CtbZIP12	ATGACACTGCCGAGTCTACC	CTGTTGGTTTAACGCCCTC
CtbZIP13	GAGCAGCCTTCTTGGCTG	GCTTATTGTCCATCTGGTCGT
CtbZIP14	TAGATACTCGTCCCAAAGCG	TTGCCAACCCATCAAATCC
CtbZIP15	TCTCCGCCAGTTTCATCG	CTCTGCCTCCGTTCCACA
CtbZIP16	ACCTGCCAAAGACGCAAA	GGAGAATAAGCCTGAAACCC
CtbZIP17	ACAGCCAGATCGCGTCG	CACCGCCGCCGTAAAT
CtbZIP18	GAGCAGCCTTCTTGGCTG	TCATTATTCGCAGCATTAGC
CtbZIP19	AGCACCTAGACGAGCTTTGG	TAAGCTGGGCATTCTGTTGA
CtbZIP20	GAGAAACGAGGGAATGACG	GCCTGCTGCCAACACAAT

CtbZIP21	AGATTGCCCAGGAGGTTAGT	GGATGATGCTGTAATAGGGTG
CtbZIP22	CGTCGAGCGTAAGCATCA	CCGTAAATCTCCGCCTGT
CtbZIP23	GGTAGGCAATCGGGAAATC	AGCCTCCGATTCCAAACC
CtbZIP24	CGGCTGTTGCTTTGACTAGG	CCTTACCTGGTCCCTTGAGAT
CtbZIP25	AGAACTCGGTGATTAGGGCT	GTTTGTGCAATGGCTGGT
CtbZIP26	GGAGGTGCGAGTGAAAGAAT	CGGATAACCGTTTGGTAAAC
CtbZIP27	TGCCGCAATCTATCCACAT	CATTCCCTATGGACATTGCC
CtbZIP28	GGAGCCGTTACTGCGATTA	GTCTGGCACTTCCTGCTTAC
CtbZIP29	ATGGTGTGGTAGGATTTGGG	CATCCTTCTTTGCCTCTGCT
CtbZIP30	GCGGAAACTCGCTCCAA	CGCCAGCAGGTCGTTCA
CtbZIP31	GAGGATACGGAAGAAGAAACAC	AACCGAGCCCTAAGAACC
CtbZIP32	GATAAATCCACCAAAGAGGCA	TAAGGCTGAAACCCAGTCCA
CtbZIP33	AAGTGTTTGGATGGGTCTGC	CCATTCCTTTGGCACTGCT
CtbZIP34	CAATGCTCAACGGTCACG	CTCCAGAGACAAATAAATACGC
CtbZIP35	AAGCGGAGGAGGCACTG	TGCACAAGTCATCTGGGAGT
CtbZIP36	TCGGTGCTGAAGGCTCG	GCCGCCGTACATTTCCT
CtbZIP37	ATGCCACCATCTTCTTTCCA	TTCGTCGGAAGTACTACTGGAG
CtbZIP38	GACTAAAGAGACTACCAACCTGCT	AAAGAGACTACCAACCTGCTTTC
CtbZIP39	TTCTCCAAATACCCTTCATCTC	TTGTTTCCTTACCCTTGACCT
CtbZIP40	GCGAAGTGTTTCTTGGTGATT	CCATTTGCAAATCCGTCG
CtbZIP41	TGTTGACCCAAAGCGTGC	CAGCTGAGCGGATAATGATGT
CtbZIP42	TCAACGTGCTTCATCCGA	CATTAGCCGCCTCCAAA

CtbZIP43	CATTGTCGCCACGGGTA	GCCTCTTGATGAGCGTCTT
CtbZIP44	GGAACGGTGAGTTTACGGG	CAGCAGACTGGCGATTGG
CtbZIP45	GAGCAGTGGGTTGAAGAA	GAGATTCGCATAGTGGTT
CtbZIP46	CTAAGCACGGCTAACCTCCT	AGGGCTCGCGTCCAATA
CtbZIP47	TGGTGGATGATGAGAAGAAGAT	GATTTGGTCGTTGAGATCCTTC
CtbZIP48	TGCAGTTTCAAATCCCACC	TCTCGGAATAACCCGCTT
CtbZIP49	TGAACCTAAATAACTCGCCAC	TCCGTTTCCGTTCGTTTA
CtbZIP50	GAAGTGGAAAGGCTGAGAATG	GGTATTCCGTTTGTTTCTGGT
CtbZIP51	TCTTCCAGAGGGATACAAATG	CTTTCCACTTCCTGCTTCAG
CtbZIP52	TTCTACCCACAGTCTTCATCTCT	GTTCGATATCATCCGTCTTTG
